# The complete chloroplast genome sequence of a *Citrus australasica* cultivar (Rutaceae）

**DOI:** 10.1080/23802359.2021.2008842

**Published:** 2021-12-15

**Authors:** Qin-Nan Cai, Hong-Xin Wang, Da-Juan Chen, Xiu-Rong Ke, Zhi-Xin Zhu, Hua-Feng Wang

**Affiliations:** a Hainan Key Laboratory for Sustainable Utilization of Tropical Bioresources, College of Tropical Crops, Hainan University, Haikou, China; b Zhai Mingguo Academician Work Station, Sanya University, Sanya, China

**Keywords:** *Citrus australasica*, Rutaceae, plastome, genome structure

## Abstract

*Citrus australasica* (F. Muell.) Swingle belongs to the family Rutaceae. *Citrus australasica* is native to eastern Australia and southeastern New Guinea, and is mainly concentrated in a small region of northern New South Wales and tropical rainforest areas in southern Queensland. The complete plastome length of *C. australasica* is 160,335 bp, with the typical structure and gene content of angiosperm plastids, including a 26,592 bp repeat B (IRB) region, 26,952 bp IRA, 87,678 bp large single copy (LSC) region and 18,756 bp small single copy (SSC) region. The plastid contains 135 genes, including 89 protein-coding genes, 37 tRNA genes, and 8 rRNA genes. The total G/C content of the *C. australasica* plastome is 38.4%. The complete plastome sequence of *C. australasica* will provide useful resources for conservation genetics research of this species and phylogenetic research of Rutaceae.

## Introduction

*Citrus australasica* belongs to oranges in the family Rutaceae. The species mainly grows in tropical rain forest areas. *Citrus australasica* has short plants with small branches and dense leaves, blooming and fruiting many times a year. Individuals are excellent material for potted plants and can be used as hybrid parents to cultivate new varieties (Zhang et al. 2008). Oranges produce a single embryo, and are easy to obtain hybrid seedlings; plants are short and have strong drought resistance, which can be used to cultivate dwarf and drought-resistant rootstocks (Rivarola et al. 2011). The species also has a certain medicinal value and a therapeutic effect on people with weak gastrointestinal function. Therefore, we report in this study the full plastome of*C. australasica* (GenBank accession number is MZ929414.1) to improve the quality of Rutaceae related collections, medicinal applications and phylogenetic research. In this study, *C. australasica* specimens were collected from Hangzhou Botanical Garden. The voucher specimen (voucher code: D J Chen, X R Ke, A8, HUTB) and associated DNA were deposited in the Herbarium of the Institute of Tropical Agriculture and Forestry (code of herbarium HUTB), Hainan University, Haikou, China.

This experiment was reported by Zhu et al. (2018). The cleaned sequencing data of the *Citrus* plastome was assembled using MITObim v1.8 (le-petit-quevilly, France) (Hahn et al. 2013). Geneious R8.0.2 (Biomatters Ltd, Auckland, New Zealand) was used to annotate the plastid of NC_053572, and theannotation was corrected with DOGMA (Wyman et al. 2004).

**Figure 1.**
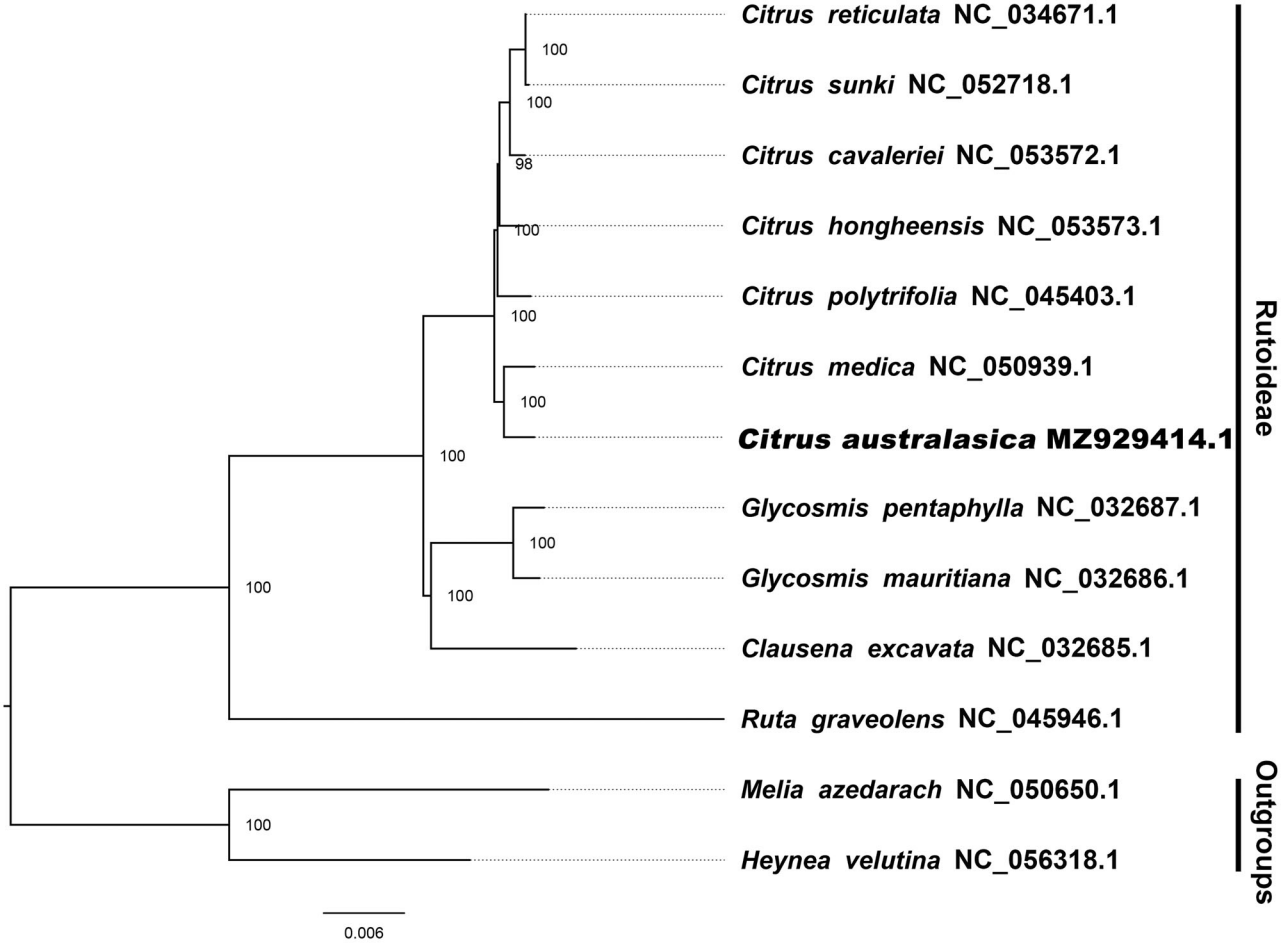
The best ML phylogeny was obtained from 13 complete plastid sequences by RAxML.

The results of this study show that the plastid length of *C. australasica* is 160,335 bp, with a typical four-part structure of angiosperms, including repeat (IRs) regions of 26,592 bp and 26,952 bp, a large single-copy (LSC) region of 87,678 bp, and a small single-copy (SSC) region of 18,756 bp. The plastid contains 135 genes, including 89 protein-coding genes (9 of which are repeated in the IRB), 37 tRNA genes (7 of which are repeated in the IRB) and 8 rRNA genes (5S rRNA, 4.5S rRNA, 23S rRNA and 16S rRNA; 4 of them are repeated in the IRB). The total G/C content of *C. australasica* is 38.4%. The corresponding values of LSC, SSC, IRA, and IRB are 36.8%, 33.1%, 43.0%, 43.1%, respectively. We used the online CIPRES portal (http://www.phylo.org/portal2/login! input.action) to establish evolutionary relationships. By inferring phylogenetic relationships based on existing data of related taxa, we found that the closest relative of *C. australasica* is *Citrus medica* (Figure 1).

The current data and analyses show that most nodes within the plastome ML tree are strongly supported. At present, the plastid sequencing of common Rutaceae plants has gradually increased and assemblies appear to be complete. This is of great significance for promoting the related protection and phylogenetic research of members of Rutaceae, and for deepening the understanding of these plants.

## Data Availability

The genome sequence data that support the findings of this study are openly available in GenBank (https://www.ncbi.nlm.nih.gov/) of NCBI with registration number MZ929414.1. Related BioProject, SRA, Bio – Sample number are PRJNA748537, SRR15498082 and SAMN20607950, respectively.
